# Distinct blood and oral microbiome profiles reveal altered microbial composition and functional pathways in myocardial infarction patients

**DOI:** 10.3389/fcimb.2025.1506382

**Published:** 2025-04-14

**Authors:** Shengnan Lei, Tuo Chen, Jianye Zhou, Linghong Zhu, Zilong Zhang, Xiaodong Xie, Xu Zhang, Ikram Khan, Zhiqiang Li

**Affiliations:** ^1^ Department of Medicine, Northwest MINZU University, Lanzhou, Gansu, China; ^2^ Northwest Institute of Eco-Environment and Resources, Chinese Academy of Sciences, Lanzhou, Gansu, China; ^3^ Department of Northwest Institute of Eco-Environment and Resources, University of Chinese Academy of Sciences, Beijing, China; ^4^ Department of Medicine, Gansu Provincial Key Laboratory of Oral Diseases Research, Lanzhou, China; ^5^ Department of Oncological Surgery, Qinghai Provincial People’s Hospital, Xining, Qinghai, China; ^6^ Department of Genetics, School of Basic Medical Sciences, Lanzhou University, Lanzhou, Gansu, China; ^7^ Department of Cardiology, General Hospital of Xizang Military Region, Lhasa, China

**Keywords:** blood microbiome, myocardial infarction, oral microbiome translocation, 16S rRNA sequencing, microbiome biomarkers, cardiovascular pathology

## Abstract

**Introduction:**

The blood microbiome, increasingly recognized as a distinct microbial niche, may originate partly from oral translocation. We systematically compared circulating and oral microbiome profiles between healthy individuals and myocardial infarction (MI) patients to identify disease-associated signatures.

**Methods:**

The current study recruited 20 participants, including 10 healthy controls and 10 patients with MI. Blood and Saliva samples were collected from each participant to analyze the association between blood and oral microbiome in MI patients using 16S rRNA gene sequencing.

**Results:**

The blood microbiome showed significantly greater alpha diversity than the oral microbiome (p<0.05), but beta diversity did not differ significantly. The blood microbiome in MI patients had higher levels of Firmicutes, Bacteroidota, Actinobacteriota, genus Bacteroides, and lower Proteobacteria, whereas the oral microbiome was dominated by Firmicutes, Bacteroidota, Veillonella, and Prevotella_7. LEfSe analysis revealed distinct blood microbial taxa-Actinobacteria in MI patients and Enterobacterales in controls. In contrast, the oral microbiota of healthy subjects was enriched in Rothia, Micrococcaceae, and Micrococcales, while no distinct taxa were associated with MI. Both blood and oral microbiomes showed significant functional pathway differences (KEGG) between groups. Additionally, microbiome signatures significantly correlated with clinical and demographic markers.

**Discussion:**

Our study demonstrates that the blood harbors a distinct microbiome characterized by specific microbial taxa and functional pathways rather than merely reflecting oral bacterial translocation. These findings suggest that the circulating microbiome may play an active role in the pathology of MI. Furthermore, we identified significant associations between these microbial signatures and clinical disease markers. This highlights the potential importance of the blood microbiome in understanding the mechanisms underlying MI and its diagnostic or therapeutic implications.

## Introduction

Acute myocardial infarction (AMI) is a common cardiac emergency associated with high morbidity and mortality. As a leading cause of heart failure and cardiac death worldwide, it remains a major public health concern (Basha et al., 2020; Li et al., 2021; Khan et al., 2022a; Khan et al., 2022b). Four out of every five cardiovascular disease (CVD) deaths are attributed to stroke or MI (Tesfaye and Hirigo, 2020). Although percutaneous coronary intervention (PCI) has reduced mortality from AMI, there is still a significant need for strategies to enhance therapeutic outcomes (Zhou et al., 2018). Myocardial infarction is a complex condition influenced by numerous factors, including lifestyle, genetics, environment, and gut microbiota, which have been extensively studied (Zununi Vahed et al., 2018). Emerging research suggests that a dysbiotic blood microbiome may also play a role in MI development, though some studies argue that blood lacks a core microbiome, with microbial translocation occurring from the oral or gut microenvironment (Vojinovic et al., 2019; Velmurugan et al., 2020; Khan et al., 2022b). To date, the role of the blood and oral microbiome in MI remains largely unexplored, highlighting the need for this study to uncover their potential contributions.

Growing evidence indicates a link between oral microbiota and AMI, with serum antibody levels against various oral pathogens showing a positive correlation with myocardial infarction risk (Lund Håheim et al., 2008). Polymerase chain reaction and microbial sequencing technologies have identified oral microbial DNA, including both periodontal and non-periodontal pathogenic bacteria, in coronary artery thrombi of myocardial infarction patients (Kwun et al., 2020). This suggests a potential link between oral bacteria and the formation of coronary artery thrombi in AMI. Disruption of oral microbial ecology leading to periodontitis has been documented to increase the risk of AMI (Li et al., 2024). Additionally, multiple studies have highlighted the diagnostic and prognostic potential of blood microbiome profiles in diseases like acute and chronic myocardial infarction (Khan et al., 2022c), myocardial infarction (Amar et al., 2019; Khan et al., 2022; Khan et al., 2025), hypertension (Jing et al), Cirrhosis (Cho et al), chronic kidney disease (Shah et al., 2019), and Cancer (Dong et al., 2019), However, the relationship between the blood microbiome and its possible connection to the oral microbiome in MI remains an area that requires further investigation.

Although human blood has long been considered sterile, emerging evidence suggests the presence of a transient microbiome that may play a role in health and disease (Castillo et al., 2019). A recent study revealed an increased abundance of *Ralstonia, Faecalibaculum*, and *Gammaproteobacteria* in patients with unstable angina, while *Bacteroides, Sphingomonas, Haemophilus, Serratia, Bifidobacterium*, and *Chloroplast* were more prevalent in the AMI group (Chen et al., 2024). Another study identified several cholesterol-degrading bacterial families and genera, including Nocardiaceae, Aerococcaceae, *Gordonia, Propionibacterium, Chryseobacterium*, and *Rhodococcus*, in the blood of both MI and control groups. However, their relative abundance was significantly lower in the MI group (Amar et al., 2019). Our previous study identified distinct blood microbiome patterns in MI patients, with Proteobacteria, Acidobacteria, and Alphaproteobacteria prevalent in acute cases, while Firmicutes and *Lactobacillus* were more associated with chronic cases compared to controls (Khan et al., 2022c). These findings highlight the potential of the blood microbiome in disease differentiation and early diagnosis. Building on these findings, investigating the role of the blood microbiome in cardiovascular diseases is crucial due to its significant impact on global health.

We hypothesize that MI patients exhibit distinct microbial profiles in both blood and oral environments, characterized by reduced diversity, increased pro-inflammatory taxa, and decreased protective species. These microbial alterations may contribute to disease progression and serve as potential biomarkers for MI risk. To investigate this, we analyzed and compared the blood and oral microbiomes of MI patients and healthy individuals, aiming to determine whether the blood harbors a core microbiome or if its microbial composition originates from the oral cavity. Additionally, we explored the relationship between these microbial communities and clinical indices. Our findings reveal a unique blood microbiome, distinct from the oral microbiome, suggesting its potential as a diagnostic biomarker. This study paves the way for metagenomic approaches to identify novel biomarkers and unravel the microbial mechanisms underlying MI.

## Materials and methods

### Ethics statement

This study was conducted following the Declaration of Helsinki and received approval from the Ethics Committee at Northwest Minzu University (Approval no. XBMU20230074). Written informed consent was obtained from all participants before their inclusion in the study.

### Study population

To examine the profiles of blood and oral microbiota in healthy and MI subjects, we enrolled 10 healthy individuals and 10 confirmed patients with MI from the Qinghai People’s Hospital, Qinghai Province, China. The inclusion criteria were as follows: confirmed patients with myocardial infarction and healthy controls with no history of CVD. Exclusion criteria for both groups were as follows: participants with missing data, oral diseases, diabetes, kidney disease, stomach issues, recent blood transfusions, pregnancy, inability to comply with sample collection, or use of medications affecting results (e.g., antibiotics, immunosuppressants).

### Blood sample collection

Blood samples were collected by clinically certified personnel using Vacutainer EDTA tubes, adhering to stringent hygiene protocols. The medical team, equipped with lab coats, disposable gloves, and masks, utilized sterilized reagents and materials to minimize contamination. A 5-ml blood sample was collected from each participant in the morning following overnight fasting, immediately transferred to sterile centrifuge tubes, and stored at -80°C for subsequent analysis.

### Oral health examination and saliva sample collection

All participants underwent a comprehensive oral examination by a single dentist to evaluate factors influencing the oral microbiome. None showed signs of periodontal disease, including gingivitis or periodontitis, and the prevalence of dental caries was minimal. The teeth were in good condition, with negligible wear, no visible decay, and no prior restorations. Gums were observed to be healthy-pink, firm, and tightly adhered to the teeth without any signs of bleeding or inflammation. No active oral lesions, such as ulcers, sores, or indications of oral cancer, were present. Participants demonstrated good oral hygiene, with normal tooth count and function requiring only basic care. Importantly, no professional dental cleanings had been performed in the past year, and no periodontal treatments were received in the last six months, ensuring consistent and comparable oral conditions across the study.

Before saliva collection, participants rinsed their mouths with water and closed their mouths for about a minute to minimize any contamination from microorganisms or food particles. Using sterile containers, 3 to 5 ml of saliva samples were carefully collected from each participant. Then, the samples were transferred to sterile centrifuge tubes immediately and swiftly stored at -80°C, preserving them for further analysis.

### Demographic and clinical characteristics

The data encompass various categorical variables such as gender, age, weight, height, smoking, and diabetes were obtained from the questionnaire. Body mass index (BMI) was measured by (kg/cm^2^), and systolic and diastolic blood pressure were recorded in millimeters of mercury (mmHg). Various serum parameters were assessed during screening, including Low-density lipoprotein (LDL), High-density lipoprotein (HDL), and Triglycerides (TG). These factors serve as covariates in exploratory statistical analyses, as many are recognized as potential confounders in various diagnoses related to atherosclerosis and thrombosis in CVD.

### DNA extraction and amplicon sequencing

The DNA was extracted with the TGuide S96 Magnetic Soil/Stool DNA Kit (Tiangen Biotech (Beijing) Co., Ltd.) according to manufacturer instructions. The DNA concentration of the samples was measured with the Qubit dsDNA HS Assay Kit and Qubit 4.0 Fluorometer (Invitrogen, Thermo Fisher Scientific, Oregon, USA).

The 338F: 5’- ACTCCTACGGGAGGCAGCA-3’ and 806R: 5’- GGACTACHVGGGTWTCTAAT-3’ universal primer set was used to amplify the V3-V4 region of 16S rRNA gene from the genomic DNA extracted from each sample. Both the forward and reverse 16S primers were tailed with sample-specific Illumina index sequences to allow for deep sequencing. The PCR was performed in a total reaction volume of 10 μl: DNA template 5-50 ng, *Vn F (10μM) 0.3 μl, *Vn R (10μM) 0.3 μl, KOD FX Neo Buffer 5 μl, dNTP (2 mM each) 2 μl, KOD FX Neo 0.2 μl, ddH2O up to 10 μl. Vn F and Vn R are selected according to the amplification area. After initial denaturation at 95°C for 5 min, followed by 25 cycles of denaturation at 95°C for 30 s, annealing at 50°C for 30 s, and extension at 72°C for 40 s, and a final step at 72°C for 7 min. The total of PCR amplicons was purified with Agencourt AMPure XP Beads (Beckman Coulter, Indianapolis, IN) and quantified using the Qubit dsDNA HS Assay Kit and Qubit 4.0 Fluorometer (Invitrogen, Thermo Fisher Scientific, Oregon, USA). After the individual quantification step, amplicons were pooled in equal amounts. For the constructed library, use Illumina novaseq 6000 (Illumina, Santiago, CA, USA) for sequencing.

### Bioinformatics and statistical analysis

The bioinformatics analysis of this study was performed with the aid of the BMK Cloud (Biomarker Technologies Co., Ltd., Beijing, China). According to the quality of a single nucleotide, raw data was primarily filtered by Trimmomatic (V 0.33). Identification and removal of primer sequences were processed by Cutadapt (V 1.9.1). PE reads obtained from previous steps were assembled by USEARCH (V 10) and followed by chimera removal using UCHIME (V 8.1). The high-quality reads generated from the above steps were used in the following analysis. Sequences with similarity ≥ 97% were clustered into the same operational taxonomic units (OTUs) by USEARCH (V 10.0), and the OTUs with abundance <0.005% were filtered. Taxonomy annotation of the OTUs was performed based on the Naive Bayes classifier in QIIME2 using the SILVA database (release 132) with a confidence threshold of 70%. Alpha diversity was analyzed by Chao1, Shannon, Simpson, and Abundance-based coverage estimator (ACE) indexes using the QIIME2 program (https://qiime2.org/). Beta diversity was measured by Princiipal component analysis (PCA), Principal coordinate analysis (PCoA), and Non-metric multidimensional scaling (NMDS) based on the Bray-Curtis distance matrix using the R language platform (version 4.2.1). Linear Discriminant Effect Size (LEfSe) analysis was used to assess the significant distinct taxa between healthy and patients with MI groups. A logarithmic Linear Discriminant Analysis (LDA) score of 4.0 was set as the threshold for discriminative features. The function prediction pathways of the oral and blood microbial community were predicted based on Kyoto Encyclopedia of Genes and Genomes (KEGG) by Phylogenetic Investigation of Communities by Reconstruction of Unobserved States (PICRUSt2). Moreover, to explore the dissimilarities of the microbiome among different factors, a redundancy analysis (RDA) was performed in R using the package ‘vegan’.

### Statistical analysis

Continuous variables were expressed as mean ± standard deviation. Fisher’s exact test was employed to analyze categorical variables, while the Mann-Whitney U test was used to assess differences in quantitative data. Statistical analyses were performed using Excel, GraphPad Prism, and R, with a p-value of less than 0.05 considered statistically significant.

## Results

### Baseline characteristics

A total of 40 samples were sequenced, comprising 20 blood samples and 20 saliva samples, with 10 from healthy individuals and 10 from MI patients in each site. The demographic and clinical characteristics of patients with MI and controls are presented in **Table 1**. MI patients and controls were compared according to age, sex, smoking, BMI, HBP, PD, SBP, DBP, LDL, HDL, TG, and the prevalence of diabetes. After matching, there were no statistically significant differences observed between patients with MI and control groups (*p* > 0.05).

**Table 1:** Demographics and clinical characteristics of healthy individuals and MI groups.

Variables	Healthy group	MI group	*p-*value
Age (year)	63.5 ± 13.15	65.8 ± 4.70	0.75
Sex (Male, Female)	5/5	7/3	-----
BMI kg/m^2^	23.93 ± 2.19	24.73 ± 3.77	0.57
Smoking (Yes/No)	0/10	3/7	-----
Diabetes (Yes/No)	0/10	0/10	-----
High blood pressure (Yes/No)	0/10	4/6	-----
PD (Moderate/ Mild/ Sever)	3/4/3	4/3/3	-----
Systolic BP	121.7 ± 17.48	140 ± 23.65	0.08
Diastolic BP	76.9 ± 10.09	81.8 ± 13.10	0.64
LDL	2.03 ± 0.48	2.68 ± 0.97	0.06
HDL	1.26 ± 0.26	1.15 ± 0.31	0.05*
Triglycerides	1.81 ± 1.31	1.73 ± 1.02	0.91

The data is presented as mean (±) standard deviations. Key variables include BMI (Body Mass Index), LDL (Low-Density Lipoprotein), HDL (High-Density Lipoprotein), and PD (Periodontal Disease).

### OTUs distribution

**Figure 1:**
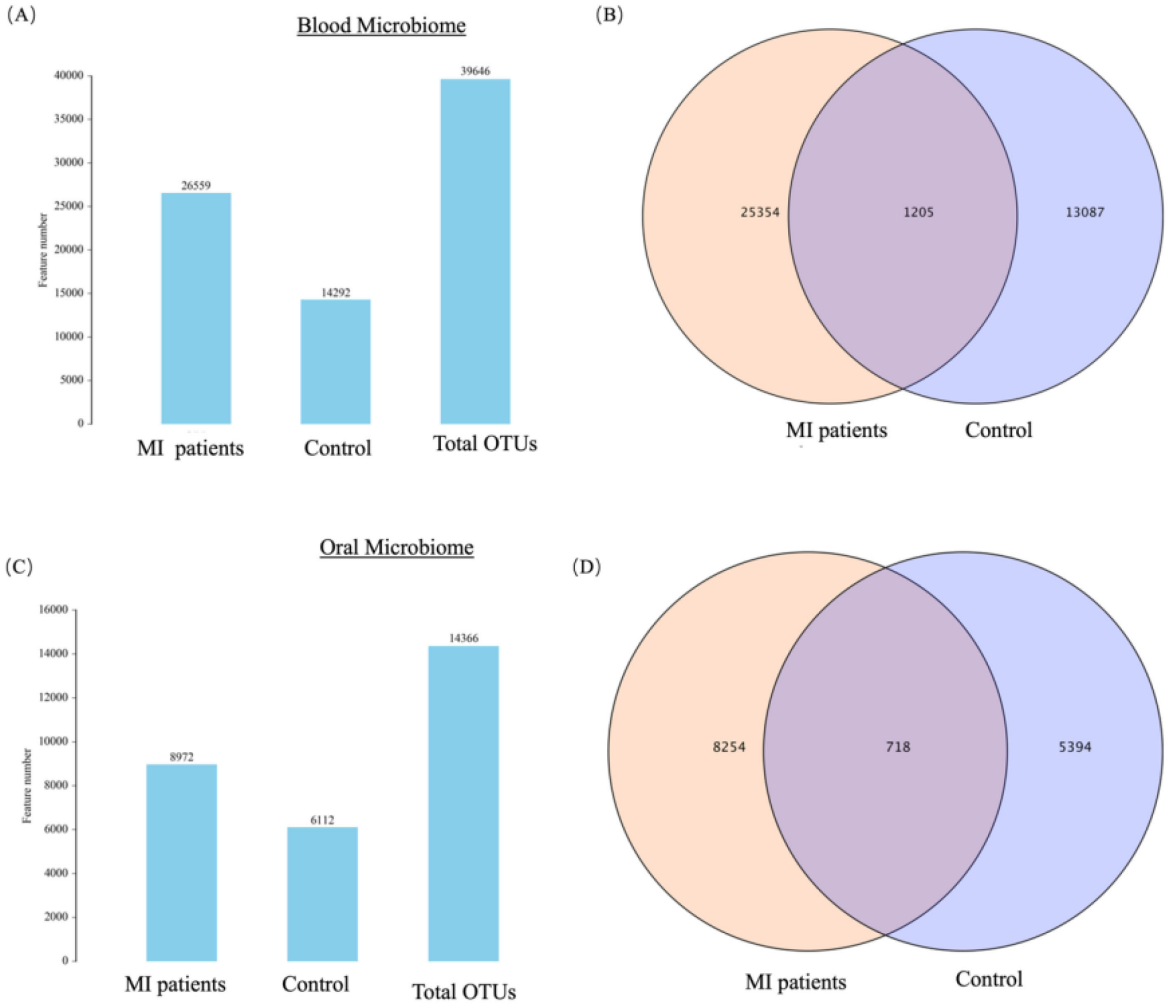
**(A)** Shows the OTUs count and distribution of the blood microbiome in MI patients and healthy controls. **(B)** Venn diagram illustrates the unique and shared OTUs in the blood microbiome of MI patients and healthy controls. **(C)** Shows the OTUs count and distribution of the oral microbiome in both groups. **(D)** Venn diagram depicts the unique and shared OTUs in oral samples between groups.

A total of 39,646 OTUs were identified from 20 blood samples (**Figure 1A**). Of these, 25,354 OTUs (63.95%) were unique to the MI group, 13,087 OTUs (33%) were unique to the control group, and 1,205 OTUs (3.03%) were shared between the two groups (**Figure 1B**). Similarly, 14,366 OTUs were identified from 20 saliva samples (**Figure 1C**), of which 8,254 OTUs (57.45%) were unique to the MI group, 5,394 OTUs (37.54%) were unique to the control group, and 718 OTUs (4.99%) were shared between the two groups (**Figure 1D**).

### Increased blood and decreased oral alpha diversity in MI

**Figure 2:**
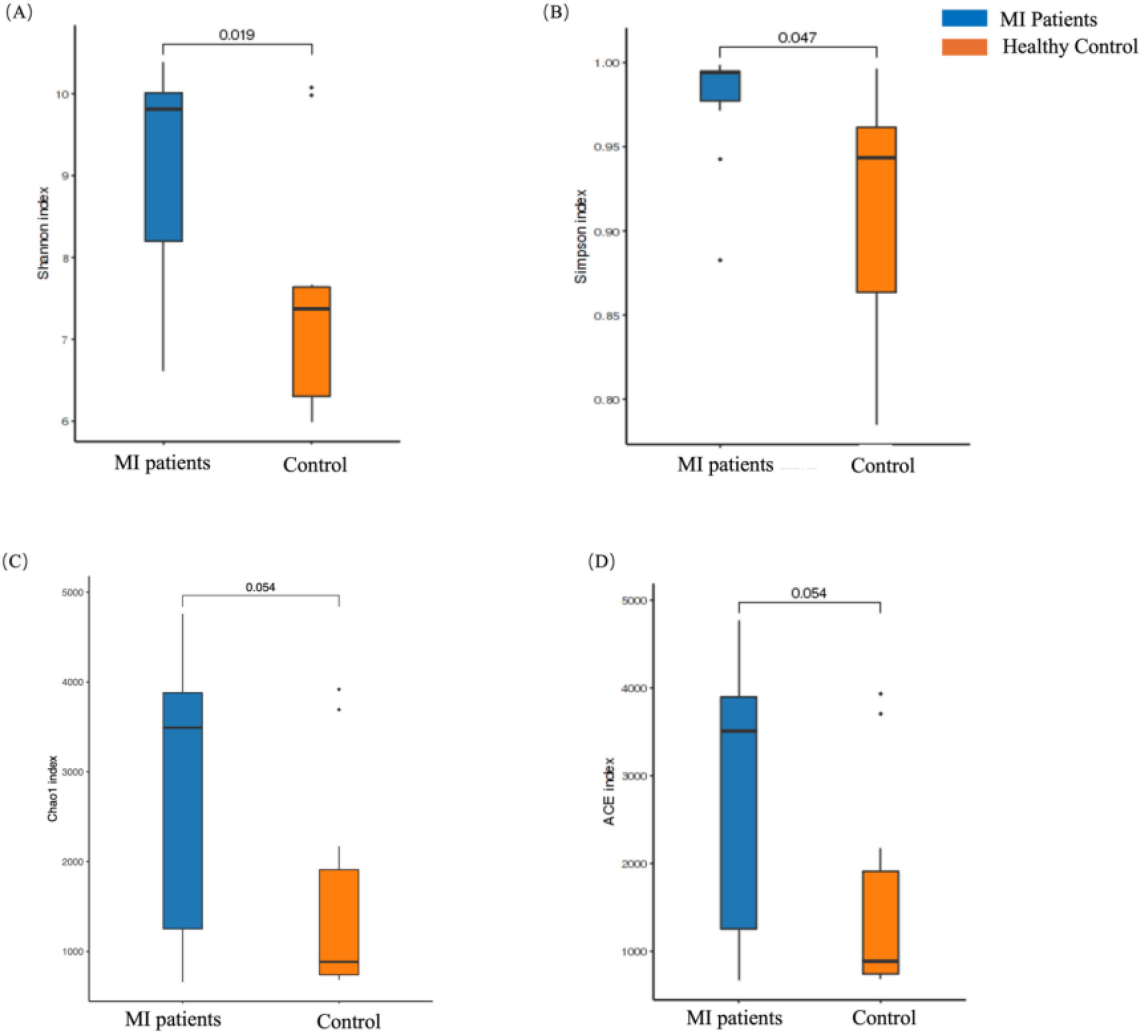
The alpha diversity analysis of the blood microbiome between MI patients and healthy groups. **(A)** The Chao1 index represents species richness in patients and healthy groups, **(B)** the Shannon index highlights differences in microbial composition between the two groups, **(C)** the Simpson index illustrates diversity in microbial composition, and **(D)** the ACE index reflects species richness between patients and healthy individuals.

The alpha diversity of blood and oral microbiome were measured using Chao1, Shannon, Simpson, and ACE indexes between both groups. A significantly higher alpha diversity was observed in the blood microbiome across all indexes, such as Shannon (*p* = 0.01; **Figure 2A**), Simpson index (*p* = 0.04; **Figure 2B**), Chao1 (*p* = 0.05; **Figure 2C**), and ACE index (*p* = 0.05; **Figure 2D**) between MI and the control group. Overall, these findings show significant differences in the alpha diversity of the blood microbiome between both groups, which might have an association with MI.

**Figure 3:**
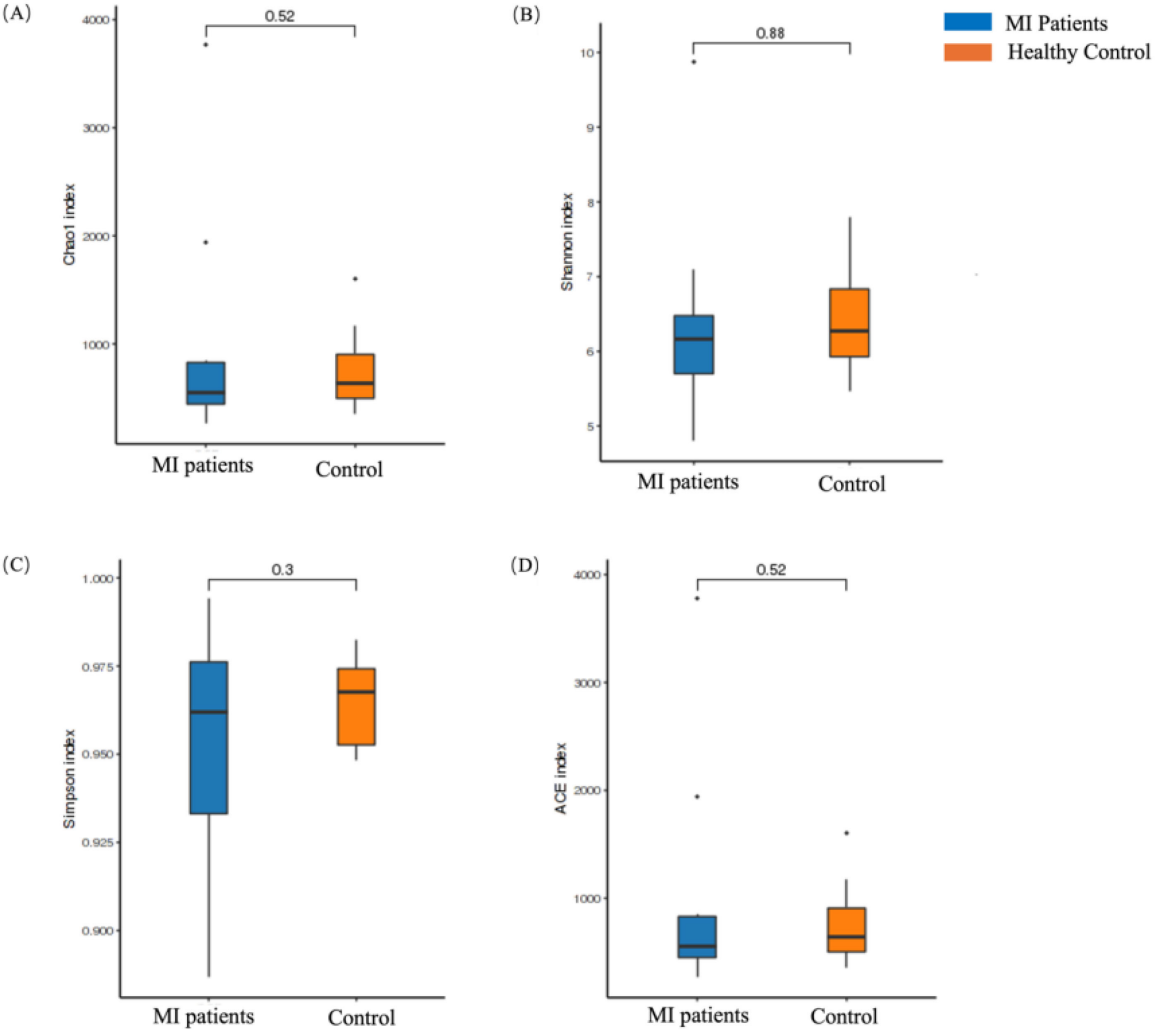
Alpha diversity analysis of the oral microbiome between MI patients and healthy groups. **(A)** Chao1 index shows species richness between patients and healthy groups, **(B)** Shannon index depicts differences in microbial composition between the two groups, **(C)** Simpson index shows differences in microbial composition between the two groups, and **(D)** ACE index depicts species richness between patients and healthy groups.

In contrast, the alpha diversity of the oral microbiome showed an opposite trend. No statistically significant differences were found in the Chao1 (*p* = 0.52; **Figure 3A**), Shannon (*p* = 0.88; **Figure 3B**), Simpson (*p* = 0.3; **Figure 3C**), and ACE (*p* = 0.52; **Figure 3D**) indexes between MI and control groups.

### Beta diversity analysis between both groups

**Figure 4:**
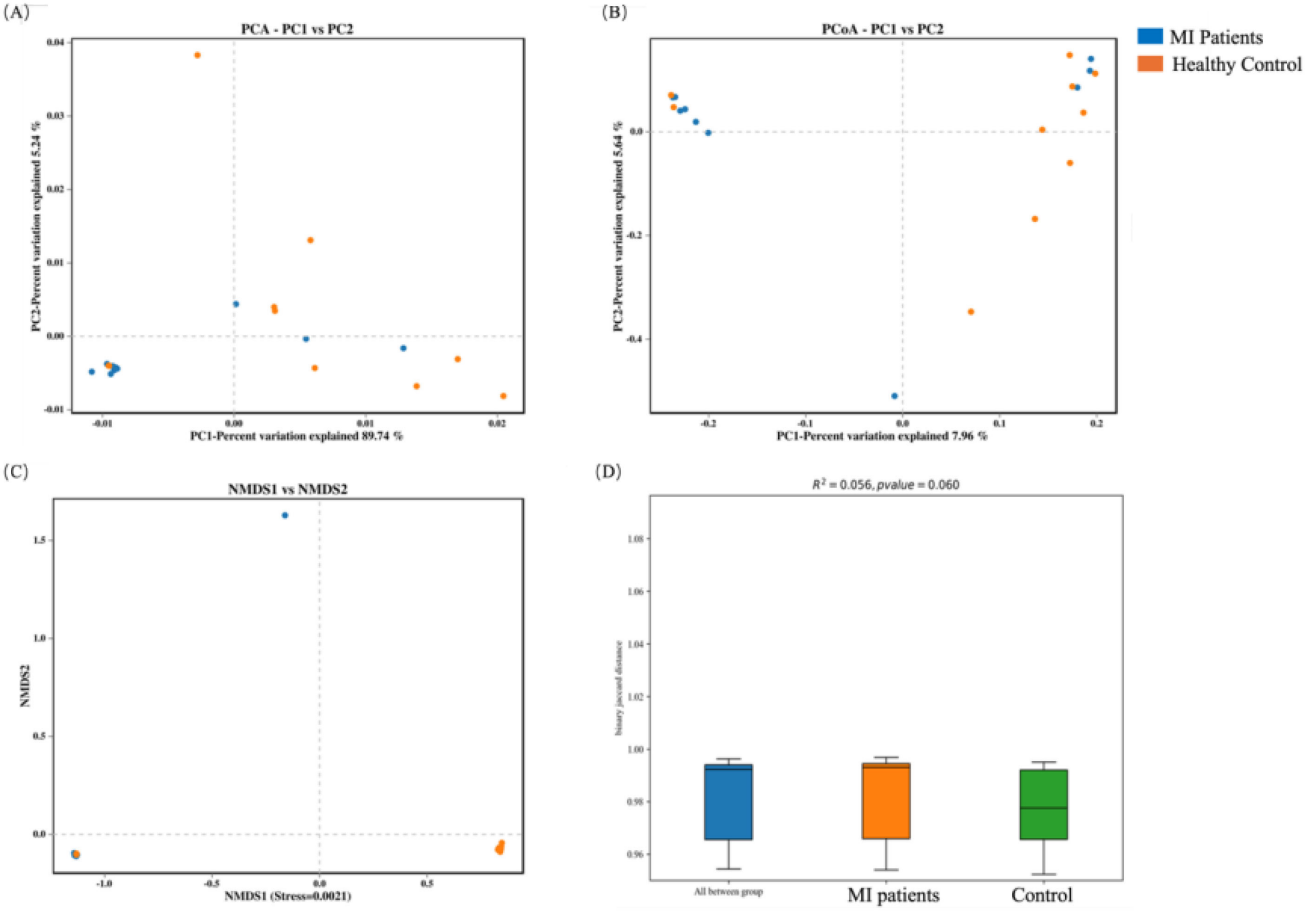
Beta diversity analysis of the blood microbiome between MI patients and healthy controls: **(A)** PCA identifies major variance patterns, **(B)** PCoA visualizes composition differences via distance matrices, **(C)** NMDS represents microbial community dissimilarities, and **(D)** PERMANOVA tests for statistical significance between groups.

In order to assess the variation in microbial community composition between the two groups, beta diversity analysis was conducted. First, the blood microbiome was assessed based on principal component analysis (PCA), principal coordinate analysis (PCoA), and Non-metric multidimensional scaling (NMDS) distance similarity metrics. No statistically significant differences in PCA (**Figure 4A**), PCoA (**Figure 4B**), and NMDS (**Figure 4C**) were observed in bacterial composition between the two groups, which was further confirmed by PERMANOVA analysis (R^2^ = 0.056, *p* = 0.06; **Figure 4D**).

**Figure 5:**
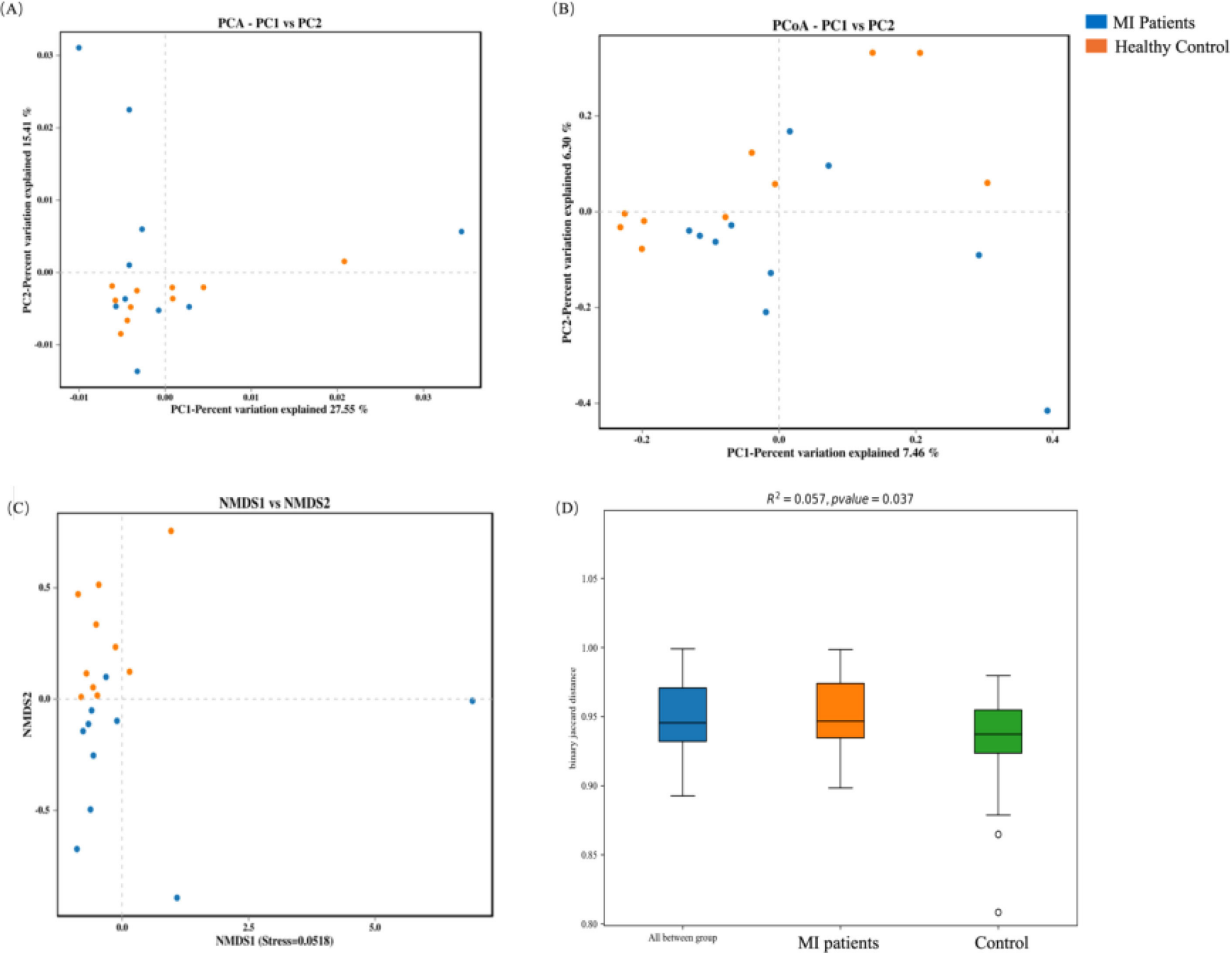
Beta diversity analysis of the oral microbiome between both groups: **(A)** PCA captures major variance patterns, **(B)** PCoA visualizes composition disparities via distance matrices, **(C)** NMDS represents microbial community dissimilarities, and **(D)** PERMANOVA assesses statistical significance between groups.

In contrast, the beta diversity of the oral microbiome showed an opposite trend. Significant differences were observed in PCA (**Figure 5A**), PCoA (**Figure 5B**), and NMDS (**Figure 5C**) indexes between MI and control groups, which was further validated by PERMANOVA analysis (R^2^ = 0.057, *p* = 0.037; **Figure 5D**).

### Taxonomic distribution of microbial taxa in MI

**Figure 6:**
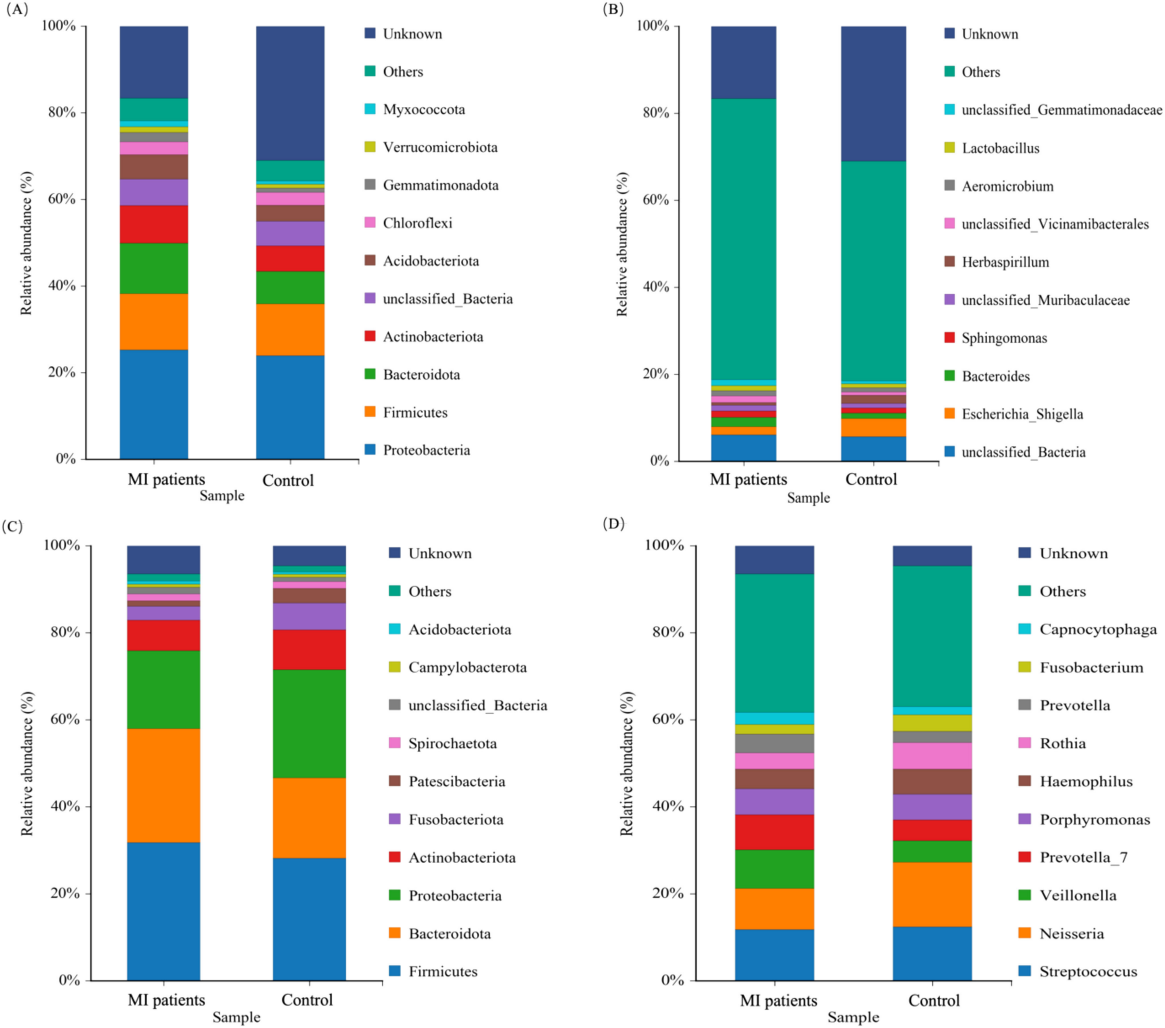
The relative abundance of the blood and oral microbiomes between MI patients and healthy groups. **(A)** Shows the dominant microbial phyla in blood samples between the two groups. **(B)** Illustrate the abundant genera in the blood between MI patients and control groups. **(C)** Shows predominant bacterial phyla present in oral samples between both groups. **(D)** Depicts the abundant genera in the oral microbiota between MI patients and control groups.

We subsequently aimed to characterize the microbial profiles associated with MI. The phylum Firmicutes was the most prevalent, accounting for 20.73% in the MI group compared to 17.53% in the control group, followed by Bacteroidota (16.79% vs. 12.19%) and Actinobacteria (11.97% vs. 8.43%). In contrast, the phylum Proteobacteria was depleted in the MI group, constituting 21.11% compared to 24.33% in the control group (**Figure 6A**). At the genus level, *Bacteroides*exhibited the highest prevalence, with a relative abundance of 11.53% in the MI group and 7.31% in the control group (**Figure 6B**). The depleted levels of Proteobacteria in MI patients could reflect altered systemic microbial transport mechanisms, while MI-enriched *Bacteroides* may indicate heightened inflammatory responses.

The phylum Firmicutes was found to be the most prevalent, constituting (36.28% vs. 32.40%), and Bacteroidota (30.17% vs. 26.12%) of the oral microbiota in the patients with MI and control groups, respectively (**Figure 6C**). At the genus level, *Veillonella* (7.97% vs. 3.91%) and *Prevotell_7 (*6.2% vs 3.1%) exhibited the highest prevalence in the MI group than in the control group (**Figure 6D**). These findings highlight distinct microbial landscapes in the blood and oral microenvironments in MI patients.

### Distinct blood and oral taxa between both groups

**Figure 7:**
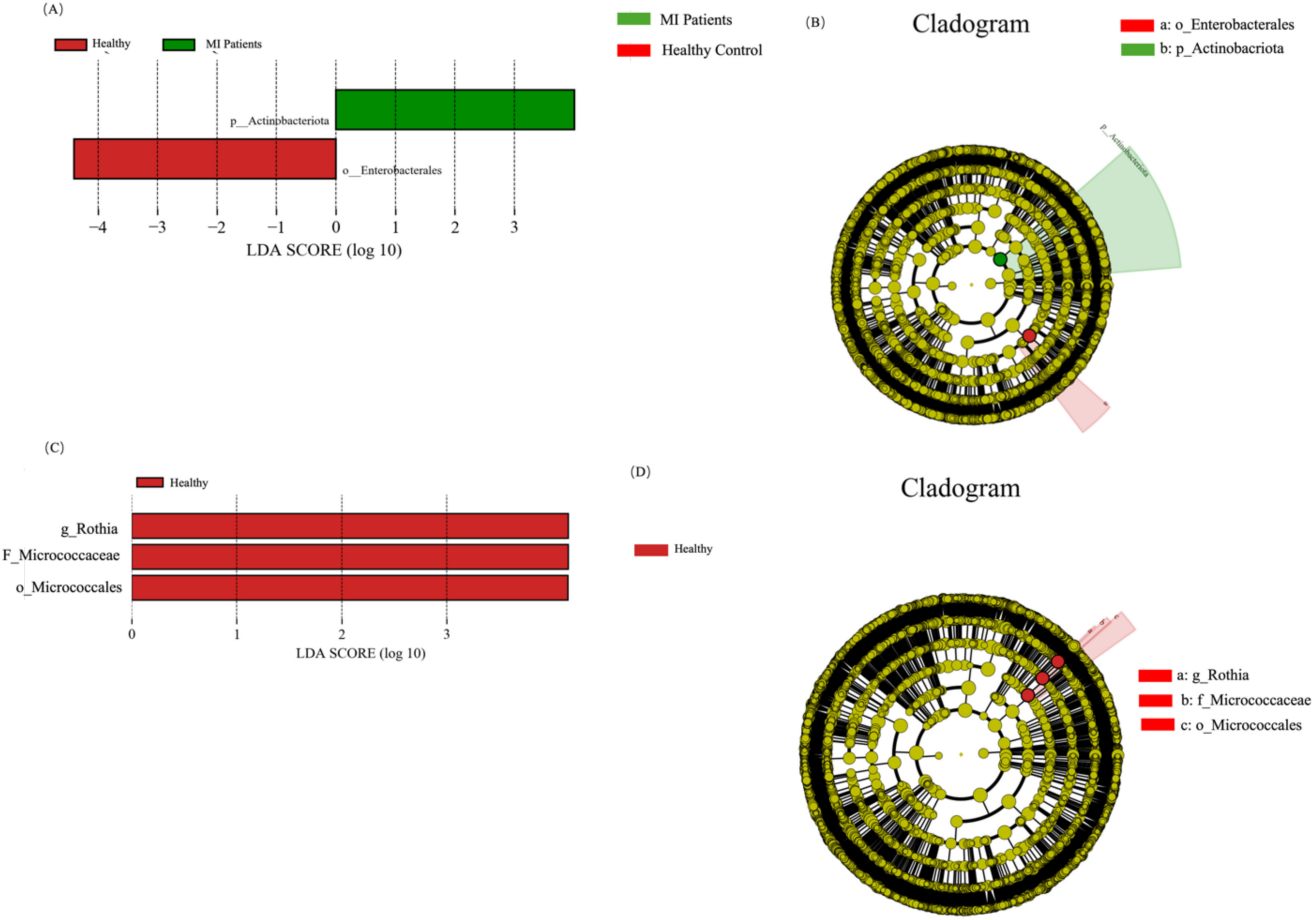
LEfSe analysis highlights microbial differences between MI patients and healthy controls at both blood and oral microbiome levels. **(A)** Shows differentially abundant microbial taxa in the blood microbiome, highlighting potential biomarkers. **(B)** Depicts phylogenetic relationships of enriched microbial taxa, illustrating taxonomic differences between groups. **(C)** Illustrate significantly altered microbial taxa in the oral microbiome, revealing shifts associated with MI. **(D)** Shows the phylogenetic structure of oral microbiota, providing a visual representation of taxonomic differences between MI patients and controls.

To further reveal the signature blood and oral microbiome profiles and predominant microbiota of patients with MI, we performed a linear discriminant effect size (LEfSe) analysis. A total of two differential abundance between the control and MI groups were identified. Phylum Actinobacteria in the MI group, while the order Enterobacterales was distinct in the healthy group, as shown in **Figure 7A**. The cladogram represents the phylogenetic profile of the blood microbiome across the two groups (**Figure 7B**).

In addition, three distinct bacterial taxa, namely genus *Rothia*, family Micrococcaceae, and order Micrococcales, were identified in the healthy group (**Figure 7C**), while no distinct taxa were observed in the MI group. The cladogram depicts the phylogenetic structure of the oral microbiome in both groups (**Figure 7D**).

### Functional enrichment analysis (KEGG annotated pathway)

**Figure 8:**
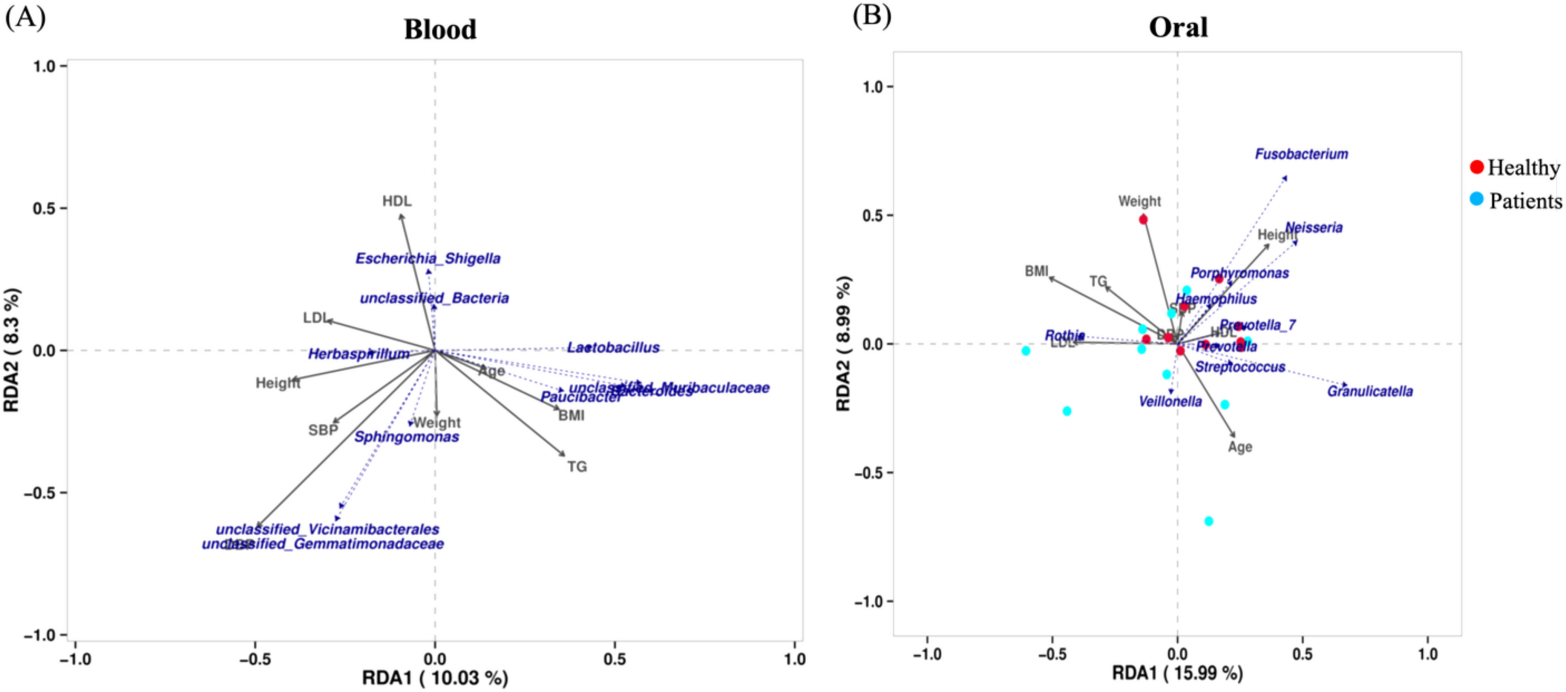
KEGG pathway analysis of blood and oral microbiomes in MI patients and healthy controls: **(A)** Shows key predicted metabolic and signaling pathways that are upregulated or downregulated in MI patients. **(B)** Depicts functional prediction pathways in oral microbial communities, highlighting metabolic shifts that may contribute to MI pathogenesis or systemic microbial translocation.

To further explore the blood and oral microbial functional dysbiosis of MI patients, we performed functional annotation and differential analyses based on bacterial profiles. We observed 32 altered pathways of the blood microbiome between both groups. Among these pathways, Biosynthesis of other secondary metabolites, Metabolism of cofactors and vitamins, Energy metabolism, Nervous system, Endocrine system, Transport and catabolism, Cancers: Overview, Global and overview maps, Aging, Transcription, GIvcan biosynthesis and metabolism, Cell growth and death, Folding, sorting and degradation, Translation, Replication and repair, Nucleotide metabolism, Amino acid metabolism, Endocrine and metabolic diseases, Metabolism of other amino acids, Neurodegenerative diseases, and Cancers: Specific types were significantly up-regulated in MI patients group, while Cellular community-prokaryotes, Infectious diseases: Bacterial, Environmental adaptation, Signal transduction, Cell motility, Drug resistance: Antimicrobial, Membrane transport, Xenobiotics biodegradation and metabolism, Lipid metabolism, Carbohydrate metabolism, and Metabolism of terpenoids and polyketides were significantly down-regulated in MI group compared to healthy, as shown in **Figure 8A**.

Additionally, the functional pathways of the oral microbiome were assessed. The functional pathways of the oral microbiome showed that the 29 pathways are altered between both groups. Among these pathways, are cell growth and death, Infectious diseases: Bacterial, Transport and catabolism, Carbohydrate metabolism, GIvcan biosynthesis and metabolism, Drug resistance: Antimicrobial, Nervous system, Metabolism of other amino acids, Environmental adaptation, Energy metabolism, Cell motility, Xenobiotics biodegradation, and metabolism, Metabolism of cofactors and vitamins, and Nucleotide metabolism pathways were significantly up-regulated in MI group, while Membrane transport, Endocrine, and metabolic diseases, Metabolism of terpenoids and polyketides, Neurodegenerative diseases, Global and overview maps, Aging, Transcription, Folding, sorting and degradation, Lipid metabolism, Replication and repair, Cellular community-prokaryotes, Biosynthesis of other secondary metabolites, Translation, Cancers: Overview, and Endocrine system were found significantly down-regulated in MI group compared to healthy (**Figure 8B**).

### Redundancy analysis of microbial taxa and MI indicators

**Figure 9:**
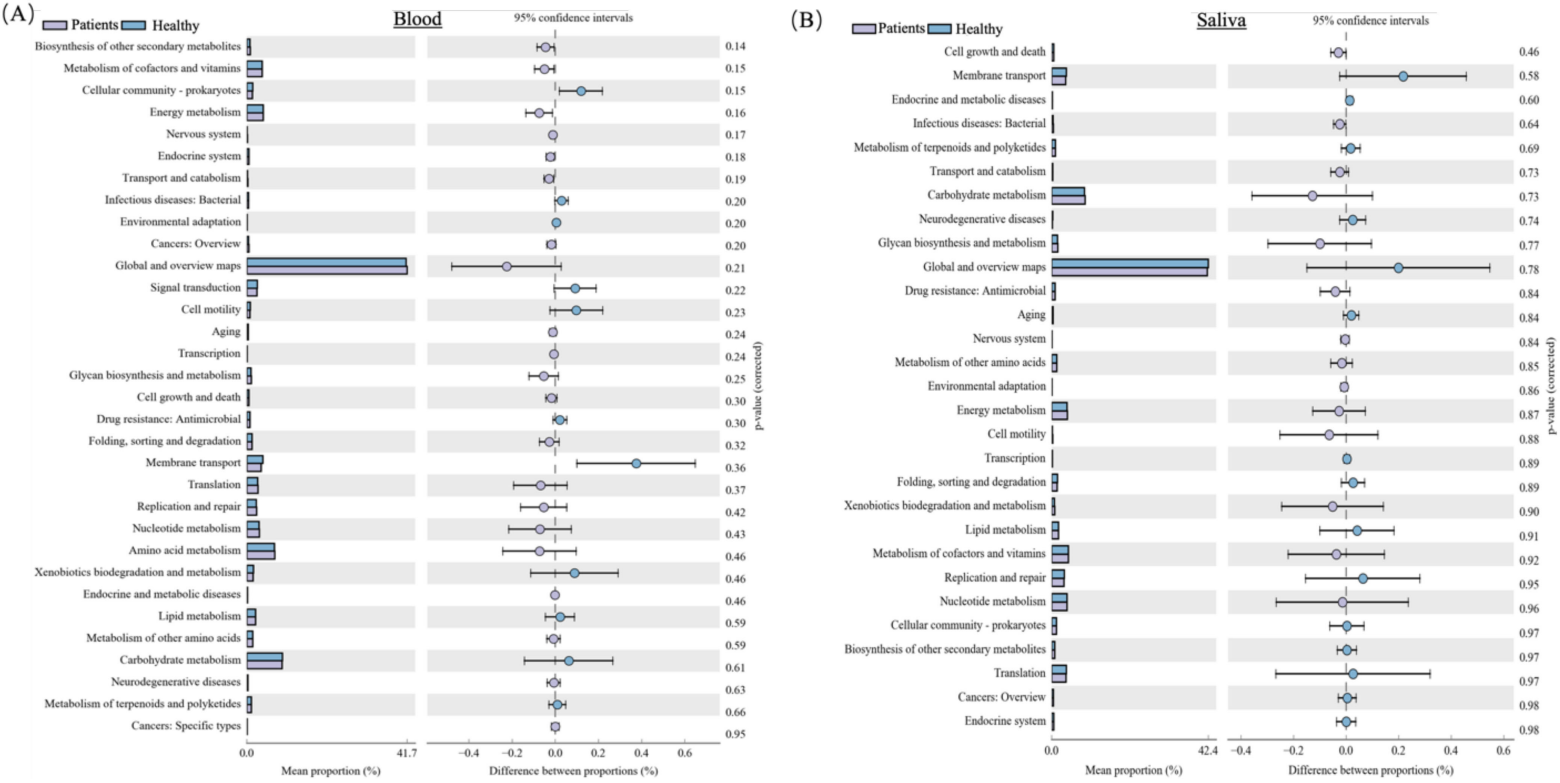
The correlation between the blood and oral microbiomes and clinical markers in MI patients and healthy controls: **(A)** Shows the association between the blood microbiome and clinical markers. **(B)** Illustrate the association between the oral microbiome and clinical markers.

The RDA analysis of blood microbiota reveals distinct microbial compositions between MI patients and healthy individuals. Several clinical parameters, including HDL, LDL, SBP, BMI, and TG, exhibit strong correlations with specific bacterial taxa, suggesting potential microbial shifts associated with MI. Notably, *Escherichia-Shigella, Herbaspirillum*, and *Sphingomonas* appear to be influenced by these clinical factors, indicating their possible role in disease progression (**Figure 9A**). The clustering of samples suggests that MI patients harbor a unique blood microbiome composition, which may serve as a potential biomarker for cardiovascular diseases. These findings further support the hypothesis that alterations in blood microbiota could contribute to MI pathogenesis.

Similarly, the RDA analysis of oral microbiota demonstrates a different microbial landscape, where no significant alpha diversity differences were observed between MI patients and healthy individuals. However, specific bacterial taxa, including *Fusobacterium, Neisseria, Porphyromonas, Veillonella, Prevotella*, and *Streptococcus*, show varying associations with clinical parameters such as BMI, weight, TG, and HDL (**Figure 9B**). This finding reinforces the notion that the blood microbiome is distinct from the oral microbiome, emphasizing the need for further research to understand their independent roles in MI pathogenesis.

## Discussion

Recently, the influence of microbiota on cardiovascular disease has gained increasing attention (Koren et al., 2011; Jin et al., 2021). Studies have identified associations between oral and gut microbiota and myocardial infarction (Pessi et al., 2013; Kwun et al., 2020). However, the relationship between blood and oral microbiota and myocardial infarction remains inadequately explored. In this study, we performed comprehensive 16S rRNA gene sequencing of blood and oral microbiota in MI patients and control subjects, obtaining detailed microbial insights. Significant differences in microbial diversity and composition were observed between the two groups. Additionally, we identified correlations between microbiota and AMI clinical parameters. These findings suggest that the blood microbiome represents a distinct microbial environment in the context of MI, highlighting its potential role in disease progression.

Under normal conditions, the mucous membranes in the mouth and digestive tract serve as barriers, preventing bacterial entry into the blood. However, when inflammation occurs in these areas, it increases membrane permeability, allowing bacterial drift into the bloodstream (Man et al., 2015; Buford et al., 2018). Increasing evidence confirms a strong association between dysbiosis of the intestinal and oral microflora and the development of CVD, highlighting the potential impact of microbial imbalances on cardiovascular health (Yang et al., 2013; Witkowski et al., 2020). Periodontitis and other oral diseases can facilitate the migration of oral flora into the bloodstream, potentially triggering conditions such as endocarditis and MI. These microorganisms may also play a role in the development of atherosclerosis, further linking oral health to CVD (Amar; Koren et al., 2011). In this study, we observed that MI patients had a higher number of OTUs in their blood than the control group, indicating a distinct blood microbiome and suggesting that its alterations may play a role in MI progression.

Our study has strengthened the connection between blood/oral microbiota and MI. Our study reinforces the link between blood and oral microbiota and MI. We observed significantly higher alpha diversity in the blood microbiota of MI patients compared to the control group. These findings are consistent with a study by Chen et al., which also reported elevated alpha diversity in acute MI patients compared to those with unstable angina, suggesting greater microbial diversity in AMI patients (Chen et al., 2024). Meanwhile, some studies have reported significantly lower alpha diversity in MI patients compared to the control group (Khan et al., 2022c; Khan et al., 2022). These findings reveal distinct shifts in blood microbiome alpha diversity between groups, highlighting a potential connection to MI and emphasizing the need for further research on its role in disease progression. Compared to blood, the alpha diversity of oral microbiota showed no significant differences between MI patients and healthy individuals. Recent studies found a significant difference in alpha diversity among AMI patients (Kwun et al., 2020; Li et al., 2024). Another study reported a significantly lower alpha diversity in the periodontal group compared to the control (Lei et al., 2024). Taken together, these findings suggest a complex and variable relationship between microbiota diversity and MI, indicating that microbial diversity may fluctuate based on disease severity, patient characteristics, or study conditions.

Previous studies have shown an enriched abundance of Proteobacteria in CCS patients compared to controls, while ACS patients exhibited a decreased abundance, indicating contrasting microbial patterns between the two conditions (Khan et al., 2022c). Similarly, elevated levels of Proteobacteria have been reported in the blood of type 1 diabetes children (Yuan et al., 2024), chronic kidney disease patients (Mair and Sirich, 2019), patients with AMI (Chen et al., 2024), and post-MI patients (Amar et al., 2019). Proteobacteria are key producers of lipopolysaccharides (LPS) (Amar et al., 2011), and the role of LPS in the etiology of atherosclerosis and MI has been documented in several studies (Carnevale et al., 2020; Hashimoto et al., 2020). Conversely, a recent study suggested that Proteobacteria regulate host arginine levels in blood circulation, where arginine has been shown to impair adipose-associated Treg suppressive function through the mTOR pathway (Meza-Perez et al., 2024). Similarly, our recent study found that Adenosine monophosphate, L-leucine, and L-arginine metabolites were downregulated in the mTOR pathway. The decreased Proteobacteria levels in MI patients may disrupt arginine metabolism, leading to altered mTOR pathway activity, which could contribute to impaired immune regulation and metabolic dysfunction associated with MI (Khan et al., 2025). Further studies are required to investigate the impact of depleted Proteobacteria abundance on arginine metabolism and mTOR pathway regulation in MI.

Furthermore, a significantly higher abundance of Actinobacteria was observed in the blood of MI patients compared to healthy controls. A previous study found that elevated Actinobacteria levels were associated with a higher risk of cardiovascular mortality, independent of factors such as age, sex, race, dialysis duration, and vascular access type (Sumida et al., 2021). Another study found elevated Actinobacteria levels in MI patients but no correlation with clinical indicators (Khan et al., 2022). Furthermore, significantly higher levels of Actinobacteria were observed in acute coronary syndrome patients compared to controls (Khan et al., 2022c). These findings suggest that alterations in the blood microbiome, specifically the enrichment of Actinobacteria and *Bacteroides* and the depletion of Proteobacteria, may serve as potential biomarkers for CVD.

Moreover, a higher abundance of *Bacteroides* was observed in the blood of MI patients compared to the control group. Higher *Bacteroides* levels in the bloodstream were observed in various diseases. For instance, Gedgaudas et al. reported an enriched presence of *Bacteroides* in the blood of portal hypertension patients (Gedgaudas et al., 2022), Yuan et al. reported in type 1 diabetic children (Yuan et al., 2024), and Zhou et al. in the blood of STEMI patients (Zhou et al., 2018). *Bacteroides* is a probiotic that is available in the human intestine, yet when it drifts to other parts of the body, it can become pathogenic, causing infections in the mouth, nervous system, and bloodstream (Amar et al., 2011). Inflammation is well-established as a key factor in atherosclerosis formation, and *Bacteroides* may contribute to acute MI by triggering an inflammatory response (Zununi Vahed et al., 2018). The enrichment of *Bacteroides* in the MI group highlights its potential role in MI pathology. However, the mechanisms by which *Bacteroides* contribute to the development or progression of MI remain unclear. Further studies are needed to elucidate the mechanisms through which *Bacteroides* influence systemic inflammation and contribute to MI pathology.

Additionally, significant distinct oral bacterial taxa in the AMI patients were reported, which include two species under *Streptococcus* (*Streptococcus oralis* subsp. *dentisani* and *Streptococcus salivarius*) and two species under *Veillonella* (*Veillonella parvula* and *Veillonella atypica*) were found to be differentially enriched in AMI patients (Li et al., 2024). Another study reported that the higher Streptococcus, Prevotella, Veillonella, Fusobacterium, and Treponema genera were higher in high-risk ischemic patients compared with that in the healthy group (Sun et al., 2023). In contrast, the current findings revealed a significantly distinct oral microbiota composition in the control group, such as *Rothia*, Micrococcaceae, and Micrococcales, although no specific bacterial taxa were uniquely detected in the MI group. These findings suggest that the oral microbiota may be influenced by environmental or dietary factors. The variability across these studies highlights the need for further longitudinal research to better understand the role of the oral microbiome in cardiovascular health and disease.

Furthermore, in the present study, we identified a significant correlation between blood and oral microbiota and clinical parameters in the MI group. Notably, blood taxa, including *Escherichia_Shigella, Lactobacillus*, *Herbaspirillum*, and *Sphingomonas*, were associated with HDL, LDL, age, BMI, TG, weight, and systolic blood pressure. Similarly, the oral taxa, namely *Fusobacterium*, *Neisseria*, *Porphyromonas*, *Haemophilus*, *Prevotella_7*, *Prevotella*, *Streptococcus*, *Granulicatella*, *Veillonella*, and *Rothia*, were correlated with clinical indexes of MI. However, these exploratory correlations highlight the need for experimental validation to establish causal relationships between blood and microbiota and clinical markers.

Despite these significant findings, this study is limited by its small sample size, which may reduce statistical power to detect subtle microbiome differences and increase the risk of false negatives. Furthermore, the limited sample diversity may introduce spurious correlations, as it does not fully capture inter-individual variability, potentially affecting the reliability of the results. Additionally, the cross-sectional design restricts the ability to determine whether microbial changes contribute to MI pathogenesis or are merely secondary consequences of the disease. To address these limitations, future research should employ longitudinal studies, allowing for a clearer distinction between causation and correlation in microbial shifts. Moreover, integrating multi-omics approaches, such as metagenomics, metabolomics, and transcriptomics, would provide a more comprehensive understanding of microbial functional pathways and their metabolic influence on MI. Expanding these studies through multicenter collaborations with geographically and ethnically diverse populations would further strengthen statistical power, ensuring broader applicability and robustness of the findings.

## Conclusion

This study pioneers blood and oral microbiome profiling in MI, laying the foundation for a systems biology approach to uncover novel biomarkers and therapeutic targets for cardiovascular diseases. Our findings reveal a distinct relationship between blood microbial taxa and MI, offering fresh insights into its systemic effects and potential treatment strategies. Interestingly, despite previous hypotheses that oral bacteria may translocate into the bloodstream and contribute to cardiovascular disease, this study did not detect oral bacterial taxa in the blood samples, highlighting the uniqueness of the blood microbiome. However, the roles of enriched Actinobacteria and *Bacteroides*, along with depleted Proteobacteria, in CVD pathogenesis remain unclear and warrant further investigation. By bridging basic research, our results pave the way for personalized diagnostic tools and targeted interventions based on individual microbial profiles. Future research should prioritize longitudinal studies to establish causality, multi-omics approaches integrating genomics and metabolomics, and validation in larger, diverse cohorts to enhance clinical applicability.

## Data Availability

The data presented in the study are deposited in the National Center for Biotechnology Information (NCBI) repository, accession number PRJNA1171562.
